# Enteropathogenic *E. coli:* breaking the intestinal tight junction barrier

**DOI:** 10.12688/f1000research.6778.2

**Published:** 2016-05-04

**Authors:** Anand Prakash Singh, Saima Aijaz

**Affiliations:** 1 Centre for Molecular Medicine, Jawaharlal Nehru University, New Delhi, 110067, India

**Keywords:** Enteropathogenic E. coli, tight junctions, paracellular permeability.

## Abstract

Enteropathogenic *E. coli* (EPEC) causes acute intestinal infections in infants in the developing world. Infection typically spreads through contaminated food and water and leads to severe, watery diarrhea. EPEC attaches to the intestinal epithelial cells and directly injects virulence factors which modulate multiple signaling pathways leading to host cell dysfunction. However, the molecular mechanisms that regulate the onset of diarrhea are poorly defined. A major target of EPEC is the host cell tight junction complex which acts as a barrier and regulates the passage of water and solutes through the paracellular space. In this review, we focus on the EPEC effectors that target the epithelial barrier, alter its functions and contribute to leakage through the tight junctions.

## Introduction

EPEC causes diarrhea in infants in developing countries and is a major cause of morbidity and mortality ^1, 2^. EPEC infection results in excessive loss of water and electrolytes from the body leading to dehydration and death ^3^. However, the underlying molecular mechanisms are not completely understood. EPEC has been reported to disrupt the ion transporters and channels as well as tight junctions in intestinal epithelial cells leading to rapid onset of diarrhea ^4, 5^. EPEC directly injects virulence factors into the host cells which target multiple signaling pathways and some have been linked to tight junction disruption ^6^. In this review, we focus only on the EPEC effectors reported to be involved in the disruption of tight junctions.

## Discussion

EPEC attaches to the apical surface of the intestinal epithelial cells and effaces the microvilli causing localized lesions ^7, 8^. EPEC uses a type three secretion system to inject at least twenty five effector proteins into the host cells ^9^. Many of the effectors are encoded by genes located on a pathogenicity island called the locus of enterocyte effacement (LEE) although non-LEE encoded effectors have also been identified ^10^. Intimate attachment of the bacterium to the host cell is facilitated by the effector, Tir (Translocated intimin receptor) which is inserted into the host plasma membrane where it serves as a receptor for intimin, the outer surface protein of EPEC ^11^.

Recent studies have revealed that the rapid onset of diarrhea in EPEC infections is a result of increased intestinal secretion as well as reduced absorption of ions and solutes ^5^. EPEC uses several effectors to disable the ion transport systems in host intestinal cells. The EPEC effector EspF inactivates the sodium-D-glucose co-transporter SGLT-1 through co-operative actions of other EPEC effectors Map, Tir and intimin and also inhibits the activity of the Na ^+^/H ^+^ exchanger NHE3 ^12, 13^. Map and the effector NleA interact with Na ^+^/H ^+^ exchanger regulatory factor 2 (NHERF2) altering its function ^14^. The net result of these effector actions is reduced uptake of Na ^+^ into the cells. EPEC also inhibits Cl ^-^ absorption in intestinal epithelial cells by downregulation of the Cl ^-^/HCO _3_^-^ exchanger SLC26A3 also known as DRA (downregulated in adenoma) ^15, 16^. This process is mediated by the effectors EspG1 and G2 in a microtubule-dependent manner as disruption of the microtubules results in a decrease in the levels of DRA at the apical surface of host cells ^16^. These data indicate that EPEC increases the endocytosis of DRA and also prevents its recycling to the apical surface by inhibiting exocytosis ^16^.

In addition to their effects on ion absorption, EPEC effectors also disrupt host cell functions by cytoskeletal reorganization, mitochondrial dysfunction, protein transport defects, suppression of immune responses and epithelial barrier disruption ^9^. Since the barrier function is regulated by the intestinal epithelial tight junctions, EPEC-mediated disruption of this complex also contributes to the onset of diarrhea.

Tight junctions seal adjacent epithelial cells and act as (i) a gate that selectively regulates the passage of ions and solutes through the paracellular space and (ii) a fence that prevents the intermixing of apical and basal plasma membrane proteins thereby maintaining cell polarity ^17, 18^. The tight junction complex consists of transmembrane proteins, adaptor proteins, small GTPases, kinases, phosphatases, transcriptional and post-transcriptional regulators ^17, 18^. The transmembrane proteins regulate cell-cell adhesion and are linked to the cytoskeleton through the adaptor proteins ^17, 18^. Paracellular permeability is regulated by the transmembrane proteins belonging to the Marvel-domain containing protein family (occludin, Tricellulin/MarvelD2 and MarvelD3) as well as by members of the claudin family ^17, 19^. Additionally, permeability is regulated by tight junction-associated guanine-nucleotide exchange factors for Rho GTPases through the modulation of the actin cytoskeleton ^17– 19^.

Leakage through tight junctions occurs in diarrhea and the EPEC effectors EspF, Map, EspG1/G2 and NleA have been implicated in the disruption of host cell tight junctions ^20– 22^.

EspF is a multifunctional effector which disrupts the tight junction barrier ^20^. The N-terminus of EspF contains mitochondrial and nucleolus targeting sequences which direct EspF to the mitochondria and nucleolus respectively where it alters their functions ^23^. The C-terminus of EspF contains three proline rich repeats with binding sites for eukaryotic sorting nexin 9 (SNX9) and neuronal Wiskott-Aldrich syndrome protein (N-WASP) ^23^. Binding of EspF with SNX9 is required for its recruitment to the plasma membrane but is not sufficient for tight junction disruption as EspF mutants deficient in SNX9 binding also disrupt tight junctions ^24^. Binding of EspF with N-WASP triggers the activation of the Arp2/3 complex leading to actin polymerization ^24^. EspF from the rabbit EPEC strain E22 has been shown to bind actin and recruit the tight junction proteins ZO-1 and ZO-2 into actin pedestals ^25^ while EspF from the mouse EPEC strain *C. rodentium* is involved in the internalization of claudin-1, -3 and -5 *in vivo* ^26^ causing tight junction disruption.

The EPEC effector Map cooperates with EspF in the disruption of tight junctions ^20^. Like EspF, Map is also targeted to the mitochondria where it alters mitochondrial functions ^20^. Map activates the Cdc42 GTPase by functioning as its GEF (guanine-nucleotide exchange factor) leading to the formation of transient filopodia ^27^. The C-terminus of Map contains a TRL (Thr-Arg-Leu) motif through which it interacts with EBP50 (ERM-binding phosphoprotein 50) also called Na ^+^/H ^+^ exchanger regulatory factor 1 (NHERF1) ^28^. Binding with EBP50 recruits the actin scaffold protein ezrin to this complex linking Map to the actin cytoskeleton. The precise mechanism by which Map disrupts tight junctions is not known but it likely occurs through the modulation of the actin cytoskeleton.

NleA has been reported to increase paracellular permeability by disrupting the tight junction proteins ZO-1 and occludin ^22^. NleA inhibits protein secretion from the ER (endoplasmic reticulum) to the Golgi by direct interaction with Sec24, a subunit of the coat protein complex II (COP-II) ^29^. It has been proposed that NleA inhibits the transport of newly synthesized tight junction proteins disrupting the barrier.

The EPEC effector EspG and its homolog EspG2 have been shown to increase the permeability of small tracers through the tight junctions ^21^. EspG binds the Golgi matrix protein GM130 disrupting the Golgi structure ^30^. Additionally, EspG deregulates the small GTPases Arf1/6 (ADP ribosylation factor 1/6) and Rab1 causing arrest of protein trafficking from the ER to the Golgi ^31, 32^. EspG binds p21-activated kinase at the same site required for Rab1 binding ^32, 33^ linking the microtubules to this signaling axis. Recent studies have shown that EspG1 and G2 not only disrupt the microtubules causing internalization of occludin but also inhibit the recovery of tight junctions in calcium switch experiments ^34, 35^ indicating that EspG regulates multiple signaling pathways ultimately disrupting tight junctions.

Finally, EspF and EspG have also been implicated in the internalization of aquaporins-2 and -3, leading to reduced water absorption ^36^.

## Conclusions

Coordinated actions of the EPEC effectors EspF, Map, NleA and EspG disrupt tight junctions. With the exception of EspF, which forms a complex with ZO-1/ZO-2, none of the other effectors have been reported to interact with any tight junction protein. Therefore, their role in the disruption of tight junctions and onset of diarrhea is possibly due to (i) modulation of the actin cytoskeleton (EspF, Map) or the microtubules (EspG); (ii) inhibition of protein trafficking from the ER to the Golgi (EspG, NleA); (iii) inhibition of tight junction restoration (EspG); (iv) interference with the functions of the Na ^+^/H ^+^ exchanger 3 (EspF), SGLT-1 (EspF, Map), NHERF-1, -2 (Map, NleA), DRA (EspG1/G2) and (v) deregulation of aquaporins (EspF, EspG).
