# Supplementary material for: Enteropathogenic E. coli: breaking the intestinal tight junction barrier
Source: F1000Res. 2016 May 4;4:231. Originally published 2015 Jul 13. [Version 2] doi: 10.12688/f1000research.6778.2 (PMC4870986; doi:10.12688/f1000research.6778.2)
Supplement: Peer review history

# Peer review: Referee response for version 2

**DOI:** 10.5256/f1000research.9314.r13852

**Published:** 2016-05-13

**Authors:** Pradeep Dudeja

**Affiliations:** 1 Division of Gastroenterology and Hepatology, University of Illinois at Chicago, Chicago, IL, USA

The authors have appropriately revised the manuscript and have addressed all of the concerns of this reviewer

I have read this submission. I believe that I have an appropriate level of expertise to confirm that it is of an acceptable scientific standard.

# Peer review: Referee response for version 1

**DOI:** 10.5256/f1000research.7282.r11552

**Published:** 2016-01-06

**Authors:** Fernando Navarro-Garcia

**Affiliations:** 1 Department of Cell Biology, Center for Research and Advanced Studies of the National Polytechnic Institute (CINVESTAV-IPN), Mexico City, Mexico

This is an interesting review, and I think it makes a positive review to the current knowledge about the effects of EPEC on the tight junctions. However, it will be interesting to note the observations posted by the first reviewer (Dr. Dudeja).

I have read this submission. I believe that I have an appropriate level of expertise to confirm that it is of an acceptable scientific standard.

# Response

**Published:** 2016-04-17

**Authors:** Saima Aijaz

**Affiliations:** Jawaharlal Nehru University, India

Thank you for your comments. I have now amended the review to incorporate the suggestions of Prof. Dudeja.

# Peer review: Referee response for version 1

**DOI:** 10.5256/f1000research.7282.r10659

**Published:** 2015-10-05

**Authors:** Pradeep Dudeja

**Affiliations:** 1 Division of Gastroenterology and Hepatology, University of Illinois at Chicago, Chicago, IL, USA

1. The first sentence conveys the wrong message.  EPEC does not cause 1 million deaths but as quoted reference 1, it causes an average of 79000 dealth/year.  The overall deaths due to diarrheal diseases are about 1 million/year.
2. The authors should first outline the mechanisms of EPEC induced diarrhea including recent work on NaCl absorption than focus on tight junctions.
3. The authors should refer to the recent papers on EPEC effects on tight junctions e.g. Glotfelty *et al.* Cell Microbiol. 2014

I have read this submission. I believe that I have an appropriate level of expertise to confirm that it is of an acceptable scientific standard, however I have significant reservations, as outlined above.

# Response

**Published:** 2015-10-06

**Authors:** Saima Aijaz

**Affiliations:** Jawaharlal Nehru University, India

Thank you for your comments Prof. Dudeja. We are working on addressing the issues raised by you and will incorporate them in the revised version.

# Response

**Published:** 2016-04-17

**Authors:** Saima Aijaz

**Affiliations:** Jawaharlal Nehru University, India

Thank you for your valuable comments. I have now addressed the concerns you had and have incorporated the literature and references related to Cl ^-^ absorption as well as references by Glotfelty et al. I appreciate your comments as they have helped me to broaden the focus of this review and helped to highlight the importance of the disruption of ion channels and transporters by EPEC.
